# Growth and Adult Height Attainment in Danish Transgender Adolescents Treated With GnRH Analog and Sex Hormones

**DOI:** 10.1210/clinem/dgae263

**Published:** 2024-04-17

**Authors:** Pernille Badsberg Norup, Mette Ewers Haahr, Peter Christiansen, Lise Aksglaede, Line Cleemann, Trine Holm Johannsen, Anders Juul, Katharina M Main

**Affiliations:** Department of Growth and Reproduction, Copenhagen University Hospital—Rigshospitalet, 2100 Copenhagen, Denmark; International Centre for Research and Research Training in Endocrine Disruption of Male Reproduction and Child Health, Rigshospitalet and University of Copenhagen, 2100 Copenhagen, Denmark; Department of Clinical Medicine, University of Copenhagen, 2100 Copenhagen, Denmark; Sexological Clinic, Mental Health Centre, Copenhagen University Hospital—Mental Health Services CPH, 2100 Copenhagen, Denmark; Department of Growth and Reproduction, Copenhagen University Hospital—Rigshospitalet, 2100 Copenhagen, Denmark; International Centre for Research and Research Training in Endocrine Disruption of Male Reproduction and Child Health, Rigshospitalet and University of Copenhagen, 2100 Copenhagen, Denmark; Department of Growth and Reproduction, Copenhagen University Hospital—Rigshospitalet, 2100 Copenhagen, Denmark; International Centre for Research and Research Training in Endocrine Disruption of Male Reproduction and Child Health, Rigshospitalet and University of Copenhagen, 2100 Copenhagen, Denmark; Department of Growth and Reproduction, Copenhagen University Hospital—Rigshospitalet, 2100 Copenhagen, Denmark; International Centre for Research and Research Training in Endocrine Disruption of Male Reproduction and Child Health, Rigshospitalet and University of Copenhagen, 2100 Copenhagen, Denmark; Department of Growth and Reproduction, Copenhagen University Hospital—Rigshospitalet, 2100 Copenhagen, Denmark; International Centre for Research and Research Training in Endocrine Disruption of Male Reproduction and Child Health, Rigshospitalet and University of Copenhagen, 2100 Copenhagen, Denmark; Department of Growth and Reproduction, Copenhagen University Hospital—Rigshospitalet, 2100 Copenhagen, Denmark; International Centre for Research and Research Training in Endocrine Disruption of Male Reproduction and Child Health, Rigshospitalet and University of Copenhagen, 2100 Copenhagen, Denmark; Department of Clinical Medicine, University of Copenhagen, 2100 Copenhagen, Denmark; Department of Growth and Reproduction, Copenhagen University Hospital—Rigshospitalet, 2100 Copenhagen, Denmark; International Centre for Research and Research Training in Endocrine Disruption of Male Reproduction and Child Health, Rigshospitalet and University of Copenhagen, 2100 Copenhagen, Denmark; Department of Clinical Medicine, University of Copenhagen, 2100 Copenhagen, Denmark

**Keywords:** growth, transgender, gender dysphoria, gnRH analog, estradiol, testosterone

## Abstract

**Background:**

Endogenous sex steroids influence the pubertal growth spurt and adult height. However, the impact of puberty suppression and sex steroids on growth in transgender adolescents is sparsely studied.

**Aim:**

We investigated pubertal growth, serum IGF-I and IGF binding protein-3 (IGFBP-3), and adult height of transgender adolescents receiving hormone therapy.

**Methods:**

Observational study of a national cohort (2016-2023) comprising 219 transgender adolescents <18 years of age. Treatment consisted of gonadotropin-releasing hormone agonist combined with estradiol or testosterone (adjusted to serum concentrations between 0 and +2 SDs corresponding to the gender identity).

**Results:**

Adult height was within ±2 SD for sex assigned at birth.

Most trans girls reached adult height within references of girls. For trans girls (bone age ≤15 years before treatment), a growth spurt was observed during estradiol therapy. IGF-I and height SD score (SDS) declined during oral estradiol administration (−0.13 SDS per month, *P* = .059, and −0.02 SDS, *P* = .001, respectively). We observed significantly lower adult height compared to target height for trans girls (−2.7 cm, *P* = .01) and significant differences between height SDS before treatment and at adult height (−0.35 SDS, *P* < .001).

Half of the trans boys remained short (<−2 SD) compared to references for boys, and most completed growth spurt before initiation of treatment. IGFBP-3 declined following testosterone treatment. There was a significant difference between height SDS before treatment and at adult height (−0.17 SDS, *P* < .001).

**Discussion and Conclusion:**

The minor reduction in adult height of trans girls after hormone treatment may be beneficial to some, whereas trans boys did not experience height gain.

A transgender individual experiences gender incongruence, ie, a discordance between their personal sense of gender and birth-assigned sex (1). The number of children and adolescents seeking help for gender incongruence has increased over the past decade (2, 3), and these children and adolescents seek hormone therapy (HT) at an earlier age than before (4). From 1997 to 2017, 16% of all newly diagnosed persons with gender incongruence in Denmark were below the age of 18 years (5). Since January 2016, the National Board of Health in Denmark approved an assessment and treatment program for transgender children and adolescents below 18 years of age, which was centralized in the Capital Region as highly specialized tertiary health care (6). The standard of care is based on clinical experience from Nordic and European countries, the World Professional Association for Transgender Health, and Endocrine Society guidelines (7, 8). To further facilitate personalized treatment, there is no lower age limit for initiating puberty suppression or sex steroids, when gender incongruence and gender dysphoria, ie, distress caused by this discrepancy, has been recognized by a multidisciplinary team. According to the guidelines, pubertal hormone suppression can begin at puberty stage Tanner 2 (7).

On average, natural puberty starts at 10 years of age in girls with breast development and at 11 years of age with testis growth to >3 mL in boys (9, 10). Puberty suppression with gonadotropin-releasing hormone agonists (GnRHa) is prescribed to suppress further development of secondary sexual characteristics and provide time for ongoing decision-making (7, 11).

During puberty, teenagers undergo a growth spurt, which is initiated by sex steroids. This growth spurt contributes to approximately 20% of the adult height (12). Peak growth velocity occurs approximately 2 years later in boys (13.5 years) than in girls (11.5 years), and it amounts to an average peak height velocity of 9.5 cm/year for boys and 8.3 cm/year for girls (12). Cessation of growth happens at approximately 14 to 15 years of age for girls and 16 to 17 years of age for boys. Therefore, GnRHa treatment in early and mid-puberty may affect growth and adult height in transgender individuals. The potential effect of the subsequent estradiol or testosterone treatment for transgender minors is still largely unknown. The sex steroid hormone effects on bone growth may differ between those with XX and XY sex chromosomes. Estradiol has a crucial effect on pubertal growth and skeletal maturation in both girls and boys. High doses of estradiol accelerate epiphyseal maturation (13-15).

The effects of GnRHa treatment on pubertal development, ie, body appearance and hormonal status, are reversible and provide a time window for ongoing decision-making before starting sex steroids, which exert irreversible changes (7, 16). Furthermore, treatment with GnRHa prevents development of further discordance between body appearance and gender identity, especially if started in early puberty (17). Some studies have reported an improvement for transgender teenagers concerning “body dissatisfaction,” depression, and anxiety after HT and later surgery (18-20). Body dissatisfaction includes height, as those assigned male at birth (trans girls) often wish to be shorter and those assigned female at birth (assigned female at birth, trans boys) want to become taller than their target height, which is determined by parental height.

Few studies have investigated the effect on growth and adult height of transgender adolescents. A narrative review from 2021 identified a massive knowledge gap about this topic (21). The review concluded that growth velocity typically decreased during GnRHa treatment, but the studies either included few participants who started GnRHa in early puberty and/or did not follow the teenagers until adult height was achieved (22-24). Since the 2021 review, 3 studies have reported on growth in transgender adolescents. Two studies from the same group looked into trans girls and trans boys separately and found a small decline in adult height for trans girls (25), most profound in the trans girls receiving ethynyl estradiol 100 to 200 µg (3.0 cm below predicted adult height (PAH), confidence interval (CI) [.2, 5.8]) whereas a small increase in adult height compared to predicted height (3.9 ± 6.0 cm above mid-parental height and 3.0 ± 3.6 cm above PAH) was shown in trans boys (26). Both studies found a decline in growth velocity during GnRHa and an increase during sex steroid treatment. The third study (27) found target height to be the best predictor of adult height and that adult height was not affected by puberty suppression and later sex steroids for the group who started treatment in early puberty.

With this observational study, we aim to investigate growth and adult height in a national unselected cohort of transgender adolescents under 18 years of age treated with GnRHa followed by or initiated together with estradiol or testosterone. By this, we aim to improve future counseling, optimize individual treatment, and align expectations.

## Methods

### Study Population

In Denmark, all pediatric transgender individuals receive hormonal treatment at the Department of Growth and Reproduction, Copenhagen University Hospital. This is an observational study of a total of 221 transgender adolescents below the age of 18 years who started HT between January 2016 and January 2023. Two trans males declined to participate in the research and were excluded before data collection. A total of 219 were included in this study (55 trans girls and 164 trans boys). Adult height was attained in 81.3% (178/219) at the time of data collection.

According to the Danish assessment program, all transgender adolescents underwent a comprehensive medical and psychosocial evaluation including screening for psychiatric disorders before being offered HT. We attempted to collect childhood growth data from the parents. The evaluation included an assessment of the gender identity and the degree of gender dysphoria. All were evaluated by a pediatric endocrinologist, a child psychologist, and a child and adolescent psychiatrist. All transgender adolescents met the criteria for gender incongruence (International Classification of Diseases, Tenth Revision diagnoses DZ768E since January 2017 or DF64 earlier) and showed signs of gender dysphoria before being offered puberty suppression or sex steroids.

### Treatment

GnRHa was given either as leuprorelin 11.25 mg subcutaneously or triptorelin 11.25 mg intramuscularly (2016-2023) at 12-week intervals or 22.5 mg intramuscularly (2021-2023) at 24-week intervals. Intervals were shortened in a few cases due to incomplete suppression of gonadotropins. Estradiol (17β-estradiol) and testosterone treatment were started at low doses and increased every 3 to 6 months depending on individual age and remaining growth potential to reach plasma values between 0 and +2 SD for Danish references corresponding to the gender identity and age. The administration route was chosen by the trans individuals and the physician together and could be changed at request.

Estradiol tablets were started at 0.5 to 1 mg and increased to 4 to 8 mg orally daily given as 17β-estradiol 0.5 mg, 1 mg, or 2 mg tablets. Estradiol patches containing 17β-estradiol were started at 12.5 to 25 µg/24 hours and increased to 100 to 200 µg/24 hours. In case of skin irritation by patches or personal preference, estradiol gel containing 17β-estradiol was used from 1 to 4 daily doses of 0.6 mg/mL. We advised transdermal application but respected individual preferences. Some changed between administration forms because of skin irritation or from pills to patches because of insufficient breast development.

Testosterone treatment was usually started with testosterone gel 1 dose equivalent to 10 mg testosterone and increased to typically 30 to 40 mg daily. Testosterone undecanoate injections were typically started when adult testosterone levels had been reached at 1000 mg intramuscularly every 12 weeks as standard but for some individualized to every 10 to 18 weeks. Trans girls continued GnRHa treatment parallel to estradiol. In trans boys, GnRHa was discontinued after reaching adult serum levels of total and free testosterone between 0 and +2 SD.

### Measurements

Height and weight were measured at each routine visit (every 3 to 6 months) (28). Height was measured with a wall-mounted stadiometer (Harpenden Stadiometer, Holtain Ltd., Crymych, Britain) to the nearest 0.1 cm and weight to the nearest 0.1 kg with a floor scale (MPE 250K100PM, KERN & SOHN, Balingen, Germany). Bone age was measured with a radiograph of the left hand and wrist by BoneXpert a.m. Greulich-Pyle method in those adolescents in which growth had not been completed. PAH was calculated with the BoneXpert method (29). The target height, an average of genetic potential, was calculated from the height of both parents, when data was available (n = 137, 28 trans girls and 109 trans boys), by


$$((\mathrm{Heigh}t_{\mathrm{Father}}+\mathrm{Heigh}t_{\mathrm{Mother}})/2)\pm 6.5$$

Adult height was defined as 2 consecutive visits with the same height measurement. At the first routine visit, clinicians only conducted clinical puberty assessments if they were uncertain of the adolescent's stage of puberty (n = 63, 18 trans girls and 45 trans boys). The others were considered to be Tanner stage genitalia/breast 4 or 5.

Nonfasting blood samples were drawn at routine visits and analyzed for IGF-I and IGF-binding protein (IGFBP)-3 with IDS-iSYS IGF-I (RRID:AB_2861357) and IDS-iSYS IGFBP-3 (RRID:AB_2895663) assays (Immuno-diagnostic Systems Ltd., Bolton, UK), respectively, based on chemiluminescence technology. Interassay coefficients of variations were below 8% for IGF-I and 14% for IGFBP-3. The corresponding lower limits of detection were 10 µg/L and 80 µg/L, respectively.

### Statistical Analysis

Results were analyzed with the statistical software R, version 4.3.0 (http://cran.r-project.org/). Growth velocity was calculated by GrowthXP (PC PAL, Bièvers, France) from the previous and the current height measurements. Height and weight were compared to the Danish references (28) and growth velocity to international references (30). IGF-I and IGFBP-3 were compared to local reference curves (31). To account for sex and age, height and IGF-I and IGFBP-3 serum concentrations were converted to SD score (SDS) by the Generalized Additive Model for Location, Scale and Shape according to the Danish references (28, 31), and growth velocity SDS were calculated by GrowthXP according to international references (30). We did not include Tanner stage as a confounder in any analyses, as we did not have this available for all individuals. All SDS values were calculated from the sex assigned at birth references.

The analyses regarding Tanner stage and timing of treatment were conducted for the whole cohort (n = 219). The 219 transgender adolescents were divided into 2 groups according to their sex assigned at birth (55 assigned male at birth and 164 assigned female at birth). Analyses of height trajectories, IGF-I and IGFBP-3, and adult height were performed for each group separately. Analyses during GnRHa represent monotherapy with GnRHa prior to sex steroids. Analyses regarding sex steroids estradiol or testosterone were made as a combined group for both individuals who had prior GnRHa treatment and those who did not.

In order to examine individuals with residual growth potential, 2 subgroups were created, comprising trans girls with a bone age ≤15 years (n = 13) and trans boys with a bone age ≤14 years (n = 16). Analyses of growth velocity and adult height were made in both groups. For the figure showing growth velocities, 2 negative growth velocities were set to zero, 1 in each group. For the statistical analysis on growth velocity during GnRHa, the SDS were used to take age and sex assigned at birth into account. Growth velocity after initiation of sex steroids was reported as the mean growth velocity per individual in the first year.

Trans girls were also subdivided into groups according to the administration of estradiol: oral (n = 16) or transdermal (n = 14) for the analysis of IGF-I SDS and height SDS. The remaining 25 trans girls either did not start estradiol within the study period or changed between administration types.

Height SDS before treatment and at adult height was evaluated for a subgroup taking medications known to affect growth (n = 40), including methylphenidate, selective serotonin reuptake inhibitors (SSRIs), and lisdexamfetamine.

To investigate if growth velocity SDS, IGF-I SDS, and height SDS slopes were significantly different from zero, student's *t*-test for the individual slopes was performed. The individual slopes were found by linear regression. A paired *t*-test was used to compare 2 measures from the same individual. Repeated-measurement analyses were performed as mixed method analysis with the R package LMMstar. These analyses were conducted on trans girls (n = 55) and trans boys (n = 164) to further account for the transition from GnRHa monotherapy to the addition of sex steroids for some individuals, as well as the missing data for adult height for others. The results were considered statistically significant at *P* < .05.

### Ethical Approval

The project is registered in the Capital Region of Denmark according to the European Union GDPR (P-2019-230). The project is approved by the local ethics committee (H-18050607) for the establishment of a biobank (by consent). The Danish Patient Safety Authority (case no. 3-3013-3117/1) and Center for Health, Capital Region, Team for Medical Records Research (R-23014626) approved the access to medical records, which included a waiver of consent.

## Results

### Study Population and HT

The median age at the start of treatment was 16.4 years (range 10.9-18.0). The clinical characteristics of the transgender adolescents before initiating HT and at adult height are described in Table 1. The largest part of the group examined for Tanner stage (45/63, 71%) was in late puberty (median Tanner stage G3 and B4) when initiating HT. Twelve trans girls (12/55, 22%) and 62 trans boys (62/164, 38%) started both GnRHa and sex steroids ie, estradiol or testosterone, simultaneously. The time interval between the prescription of GnRHa and sex steroids for the remaining was on average 11.4 months (range 0.4 to 50.2 months). Forty-two percent of the trans girls (23/55, 42%) and 51% of the trans boys (84/164, 51%) were taking other medications, including omalizumab due to asthma (n = 1), methylphenidate due to attention deficit hyperactivity disorder (n = 12), and other kinds of psychopharmaceuticals (n = 45).

**Table 1.** Study population

	Trans girls		n = 55	Trans boys		n = 164
Before hormone therapy						
Age (years)	16.3	(12.1-18.0)		16.5	(10.9-18.0)	
Height (cm)	173.6	(149.9-187.1)		165.6	(142.2-182.9)	
Height (SDS)	−0.3	(−1.9-2.4)		0.0	(−4.3-2.7)	
Weight (kg)	60.3	(34.9-105.3)		61.6	(38.3-116.6)	
Weight (SDS)	−0.3	(−3.5-2.8)		0.7	(−2.7-5.9)	
Tanner stage*^a^*	3	(2-5)	n = 18	4	(2-5)	n = 45
G/B 2 (n)	5			3		
G/B 3 (n)	5			5		
G/B 4 (n)	4			16		
G/B 5 (n)	4			21		
BA*^b^*	16.6	(10.0-19.0)	n = 38	16.0	(10.2-18.0)	n = 85
Trans girls BA ≤15/ trans boys BA ≤14 (years)	12.7	(10.0-14.6)	n = 13	13.2	(10.2-14.0)	n = 16
Treatment						
Only GnRHa started (n)	14	25%		19	12%	
Age (years)	14.3	(12.1-17.5)		13.8	(10.9-16.0)	
First GnRHa followed by estradiol/testosterone (n)	29	53%		83	50%	
Age for GnRHa (years)	16.3	(12.1-18.0)		16.0	(12.5-17.9)	
Age for estradiol/testosterone (years)	16.7	(14.4-18.5)		16.8	(15.0-18.9)	
GnRHa and estradiol/testosterone at the same time (n)	12	22%		62	38%	
Age (years)	17.2	(15.6-17.9)		17.0	(15.4-18.0)	
Adult height			n = 38			n = 140
Height (cm)	176.2	(163.5-187.0)		167.1	(143.0-184.7)	
Height (SDS)	−0.6	(−2.6-1.0)		−0.4	(−4.2-2.4)	
Weight (kg)	65.8	(52.9-108.4)		66.0	(46.8-118.1)	
Weight (SDS)	−0.6	(−2.5-2.8)		0.4	(−2.6-5.7)	

All values are given as median (range). Tanner stage is given as either G (genitalia) for trans girls or B (breast) for trans boys.

Abbreviations: BA, bone age; GnRHa: gonadotropin-releasing hormone agonist; SDS, SD score, based on references for the sex assigned at birth.

^*a*^Tanner stage was only evaluated when clinically relevant.

^*b*^Bone age was determined in those adolescents in whom growth had not been completed.

### Height Trajectories and Adult Height

**Figure 1.**
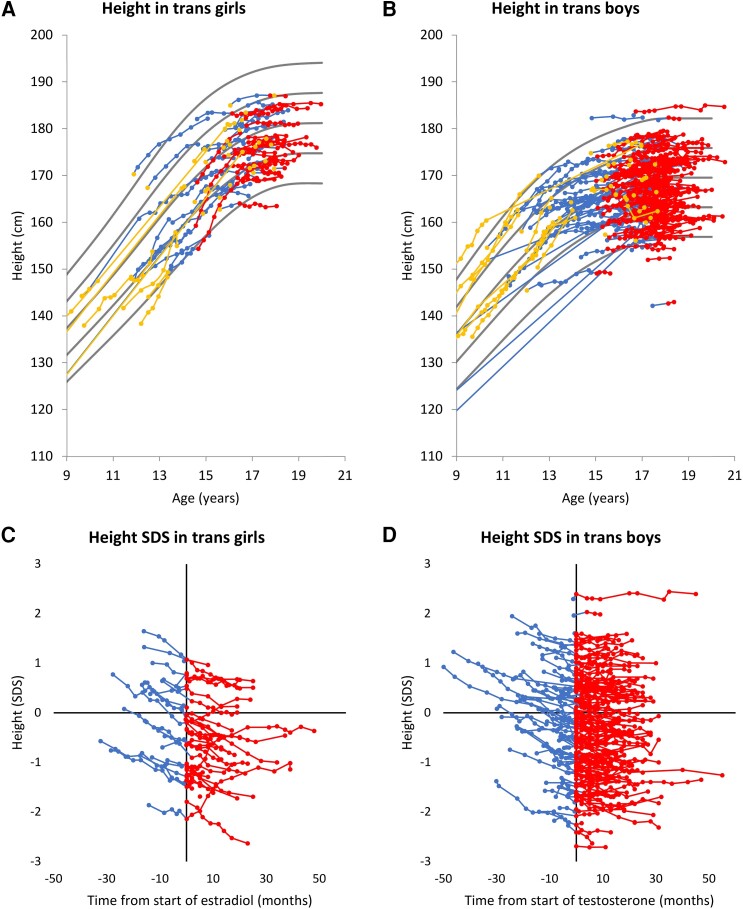
Height (cm) according to age in trans girls (A) and trans boys (B) and expressed as SDS according to days from initiation of sex steroids in trans girls (C) and trans boys (d), using Danish reference growth curves for the sex assigned at birth. Height measurements before hormone therapy (yellow), during gonadotropin-releasing hormone agonist treatment (blue), and during sex steroid treatment: estradiol or testosterone (red). Grey lines represent Danish reference ranges for the sex assigned at birth (mean, ± 1 SD and ±2 SD) (28). Abbreviations: SDS, SD score.

All trans girls, except 1, had adult heights within ±2 SD for cis boys (Fig. 1A). The height SDS trajectories indicated a decline during GnRHa treatment and an increase, for some individuals, when starting estradiol (Fig. 1C). Six (6/55, 11%) reached an adult height above +2 SD when compared with Danish references for cis girls [Supplementary Fig. S1 A (32)]. 155 trans boys (155/164, 95%) had adult heights within ±2 SD for cis girls (Fig. 1B). Height SDS trajectories during hormone therapy did not change (Fig. 1D). Almost half (78/164, 48%) of the trans boys reached adult height below −2 SD for cis boys [Supplementary Fig. S1 B (32)].

Paired *t*-tests were conducted in trans girls with available adult height, target height, and PAH [Supplementary Fig. S2 (33)]. We observed a significant difference between target height and adult height (mean difference = −2.7 cm, CI [−4.7, −.7], *P* = .01) for 18 trans girls [Supplementary Figure S2A (33)] and a near-significant difference between PAH and adult height (n = 22, mean difference = −1.5 cm, CI [−3.0, .1], *P* = .06) [Supplementary Figure S2B (33)]. For 3 trans girls who started HT with a bone age ≤ 15 years and reached adult height within the study period, we found a bigger difference between target height and adult height (n = 3, mean difference = −5.8 cm, CI [−7.8, −3.9], *P* = .006). Further, a significant difference between height SDS before HT and at adult height for trans girls was observed (n = 37, mean difference = −0.35 SDS, CI [−.49, −.21], *P* = 1.7*10^−5^) [Supplementary Figure S2C (33)].

The repeated-measurement analyses for all trans girls showed a similar decline in height SDS from before HT to adult height (−0.38 SDS, CI [−.52, −.23], *P* = 9.6*10^−6^) and a significant decline during GnRHa treatment (−0.28 SDS, CI [−.41, −.15], *P* = 1.8*10^−4^). No significant decline in height SDS was observed during estradiol treatment (−0.09 SDS, CI [−.26, .07], *P* = .26).

Paired *t*-tests were conducted in trans boys with available adult height, target height, and PAH [Supplementary Fig. S2 (33)]. We did not observe significant differences between target height and adult height (n = 87, mean difference = 0.13 cm, CI [−1.08, 1.33], *P* = .84) [Supplementary Figure S2D (33)] or between PAH and adult height (n = 70, mean difference = 0.18 cm, CI [−.09, .44], *P* = .19) [Supplementary Figure S2E (33)]. There was a significant difference between height SDS before HT and at adult height (n = 139, mean difference = −0.17 SDS, CI [−.23, −.11], *P* = 6.9*10^−8^) [Supplementary Figure S2F (33)]. For the subgroup of trans boys starting HT with a bone age ≤ 14 years, we observed no significant difference between target height and adult height (n = 6, mean difference = 3.4 cm, CI [−.5, 7.4], *P* = .077).

The repeated-measurement analyses for all trans boys showed a similar decline in height SDS from before HT to adult height (−0.21 SDS, CI [−.27, −.14], *P* = 8.7*10^−9^), a significant decline during GnRHa treatment (−0.15 SDS, CI [−.20, −.09], *P* = 6.7*10^−6^), and a nonsignificant decline during testosterone treatment (−0.06 SDS, CI [−.12; −.00], *P* = 5.8*10^−2^).

For the subgroup taking other medications known to affect growth, the average height SDS before treatment was −0.31 SDS (n = 40) compared to −0.12 SDS for the whole group (*P* = .30) and −0.37 SDS at adult height (n = 33) compared to −0.42 SDS for the whole group (*P* = .77). We observed a significant difference between height SDS before treatment and at adult height (n = 33, mean difference = −0.09 SDS, CI [−.00, −.19], *P* = .046) in this subgroup.

### Growth Velocity

**Figure 2.**
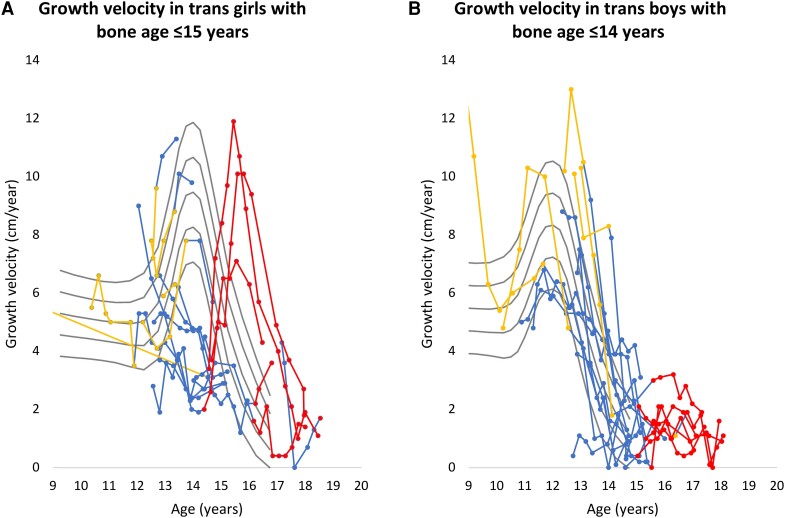
Growth velocity (cm/year) according to age in trans girls with a bone age ≤15 years (A) and trans boys with a bone age ≤14 years (B) when hormone therapy was initiated using international reference curves for the sex assigned at birth. Growth velocity before hormone therapy (yellow), during gonadotropin-releasing hormone agonist treatment (blue), and during sex steroid treatment: estradiol or testosterone (red). (A) Trans girls with a bone age ≤15 at initiation of treatment (n = 13); (B) trans boys with a bone age ≤14 at initiation of treatment (n = 16). Grey lines represent the international references for the sex assigned at birth (mean, ± 1 SD and ±2 SD) (30).

Analyses of growth velocity were conducted in the 2 subgroups of trans girls with a bone age ≤15 years and trans boys with a bone age ≤14 years at treatment start. Three trans girls had a growth spurt when estradiol treatment was initiated, which was 1 to 2 years delayed compared to cis boys (Fig. 2A). A decline in growth velocity was observed during GnRHa treatment for trans girls with a bone age ≤15 years at treatment start (n = 13, mean slope = −2.7 SDS per year, CI [−4.6, −.7], *P* = .01), but growth velocity increased when estradiol treatment was initiated (n = 5, mean growth velocity = 4.4 cm/year, range 1.3-7.2 cm/year). For the group of trans boys initiating HT with a bone age ≤14 years, we did not observe a decline in growth velocity during GnRHa treatment (n = 16, mean slope = −0.3 SDS per year, CI [−2.1, 1.4], *P* = .68) (Fig. 2B). When testosterone was initiated, they continued growing the first year (n = 7, mean growth velocity = 1.6 cm/year, range 0.9-3.1 cm/year).

### IGF-I and IGFBP-3

Overall, serum trajectories for IGF-I and IGFBP-3 concentrations for all trans girls (n = 55) and trans boys (n = 164) were within ±2 SD for the references of the sex assigned at birth [Supplementary Figs. S3 and S4 (34, 35)]. Serum IGF-I SDS seemed to be stable after initiation of GnRHa for both groups and stable after initiation of testosterone for the trans boys [Supplementary Figure S4A, C, G (35)]. An increase in IGFBP-3 after initiation of GnRHa was observed, most pronounced for the trans girls, while a decline was observed after initiation of testosterone treatment for the trans boys [Supplementary Figure S4B, D, H (35)]. Almost all trans boys ended up with a serum IGFBP-3 value between mean and −3 SD for the sex assigned at birth references.

**Figure 3.**
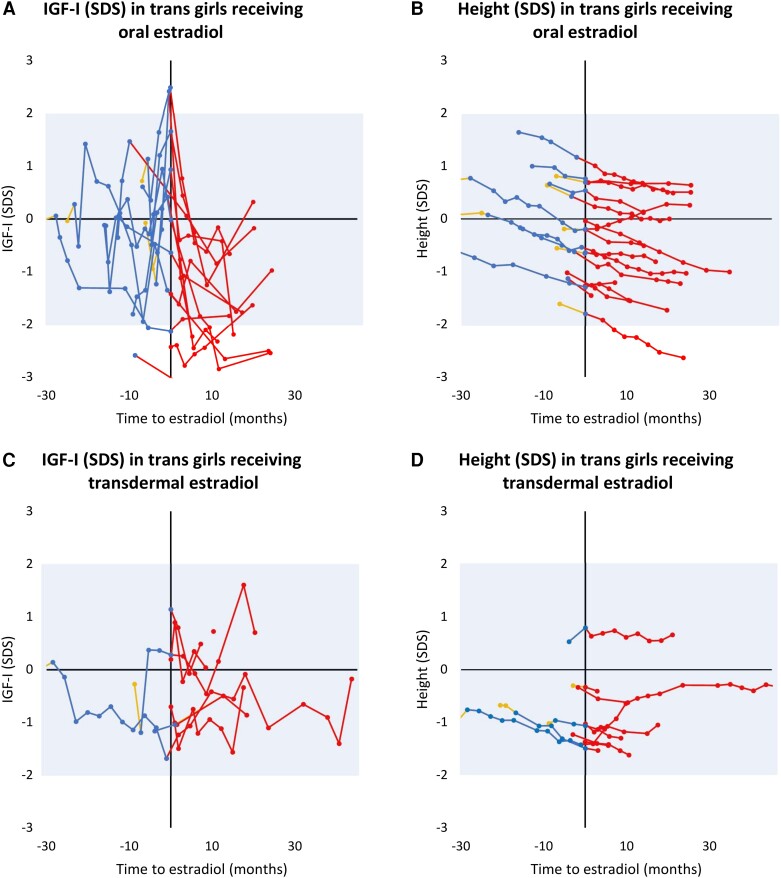
IGF-I (SDS) and height (SDS) according to time from estradiol treatment start in the subgroups of trans girls treated with oral estradiol (A and B) and transdermal estradiol (C and D). In the orally treated group (n = 16), 13 participate with IGF-I measures and all with height SDS after initiation of estradiol (A and B). In the transdermal treated group (n = 14), 7 participated with IGF-I measures and 9 with height SDS measures after initiation of estradiol (C and D). Measurements before hormone therapy (yellow), during gonadotropin-releasing hormone agonist treatment (blue), and during estradiol treatment (red). The colored areas indicate ±2 SDs according to references for local references for IGF-I (31) and Danish references for height (28) for the sex assigned at birth. Abbreviations: SDS, SD score.

For the trans girls receiving oral estradiol (n = 16), we observed a decline in IGF-I SDS after initiation that was near-significant (n = 13, mean slope = − 0.13 SDS per month, CI [−.3, .0], *P* = .059, Fig. 3A), but this was not seen in the group treated with transdermal estradiol (patches or gel, n = 14) (n = 7, mean slope = −0.01, CI [−.1, .05], *P* = .7, Fig. 3C). There was a significant decline in height SDS for the orally treated group (n = 16, mean slope = −0.02 SDS per month, CI [−.03, −.01], *P* = .001, Fig. 3B) and in the transdermal-treated group (n = 9, mean slope = −0.01 SDS per month, CI [−.03, −.00], *P* = .04, Fig. 3D). The mean age when estradiol treatment was initiated was 16.6 years for the oral (n = 16) and 17.4 years for the transdermal group (n = 14).

## Discussion

Our study provides important information about growth and adult height in a national cohort of transgender adolescents following HT with GnRHa and sex steroids. We observed a subtle reduction in the adult height of trans girls; ie, some trans girls achieved an adult height below genetic potential. Furthermore, we observed a difference between height SDS before HT and at adult height for both trans girls and trans boys. However, when reaching adult height, most transgender adolescents were within ±2 SD of Danish references for the sex assigned at birth.

GnRHa is a well-known treatment for children with central precocious puberty (CPP). In patients with CPP, GnRHa treatment can improve adult height, which does not seem to be affected if puberty is reinitiated at the average age of puberty start (36-38). However, the timing of GnRHa treatment is different for transgender adolescents. Transgender adolescents are usually treated with GnRHa at a normal age of puberty or later and with sex steroids, ie, testosterone or estradiol, much later than is physiologically typical, usually from the age of 15 to 16 years (6). This may potentially affect the growth spurt and adult height (7, 39). In addition, the effect of GnRHa treatment on adult height has previously been investigated primarily in girls, as CPP is a rare condition in boys. Therefore, only a few studies have investigated GnRHa effects on growth and adult height in cis boys (38, 40, 41).

We observed a decrease in growth velocity during GnRHa treatment for trans girls who started HT with a younger bone age but not for trans boys, probably because trans boys had achieved more of their adult height at treatment start (42). Further, GnRHa was initiated in close relation to the growth spurt for these trans girls as visualized in Fig. 2A. Similar findings have been reported in other studies (22, 24-26). A comparable decline has also been seen in cis girls with CPP (36, 37, 43). Furthermore, cis girls with CPP may improve adult height when treated with GnRHa at an early chronological and bone age (43). This was not observed in the trans girls as HT was started much later and at normal pubertal age.

In some trans girls starting GnRHa in early puberty, estradiol induced a growth spurt. We did not observe the same effect in trans boys, although some grew more than expected for the age on testosterone. The trans boys often had completed the growth spurt before initiation of HT as visualized in Fig. 2B. Estradiol has an important effect on growth in both boys and girls (14, 15). Pubertal growth and skeletal maturation are driven primarily by estrogens both via stimulation of the GH axis and thereby serum IGF-I levels, but estrogens also have a direct effect on the bones (13, 14) [for review see (14)]. Exogenous testosterone is converted to estrogens in trans boys, and estrogens will therefore still play an important role for this group. It is shown that testosterone alone, without aromatization to estrogens, can increase growth velocity in cis boys, but estrogens and the GH-IGF-I axis are still needed to reach full genetic potential (44).

Serum concentrations of IGF-I and IGFBP-3 are used clinically to examine growth disorders and to monitor treatment with GH (45). To our knowledge, no studies have reported concentrations of IGF-I and IGFBP-3 during HT in transgender adolescents. In our study, we observed stable IGF-I concentrations for the trans boys, while IGFBP-3 increased during GnRHa treatment and declined during testosterone. For the trans girls, IGF-I concentrations were stable during GnRHa but were dependent on the route of estradiol administration. We observed a visual decline in serum IGF-I in the group receiving oral estradiol, which affected the height SDS. This suggests that oral estradiol may be the preferred treatment for trans girls who do not wish to gain height. Our findings are mostly in contrast to an earlier study on transgender adults that showed a decrease in IGF-1 concentrations for trans feminine individuals after 1 year of feminizing hormone treatment but increased IGF-1 concentrations for trans masculine individuals and no changes in IGFBP-3 (46). IGF-I concentrations were stable during GnRHa, increased during testosterone, and decreased during oral estradiol treatment in transgender adolescents in the Amsterdam Cohort (25, 26), with no difference between high or regular doses of estradiol. The effect of estradiol treatment on serum IGF-I depends on the administration method (47-51), and most studies support our finding with a decline in serum IGF-I after orally administrated estradiol (52). The literature indicates either a stable concentration or an increase in serum IGF-I on testosterone treatment (53-55). Testosterone has a direct effect on the GH-IGF-I axis and through aromatization to estrogens (50, 55). Most studies observed no changes in IGFBP-3 both after estradiol and testosterone treatment.

Many of the transgender adolescents were taking other medications, including medication known to affect growth, such as methylphenidate or SSRIs. The change in height SDS during treatment in this subgroup was small and did not seem to be a primary factor for the changes observed for the entire cohort. To support this statement, a new study showed no significant changes in height SD during SSRI treatment (56), and a meta-analysis showed that the reduced growth during methylphenidate treatment is followed by catch-up growth when treatment is stopped (57).

Overall, trans girls achieved an adult height that was slightly shorter than their genetic potential. This reduction in adult height seemed to be larger for lower bone age. Our finding is similar to the finding in the Boogers et al study (25) but is in contrast to the Ciancia et al study (27), which did not find a significant difference between target height and adult height in their cohort of 22 trans girls who started HT in early puberty. Further, we observed a decline in height SDS from start to adult height. This could indicate that they were either close to finishing growth at the start or that HT influences bone age maturation. This reduction in height may be beneficial for some. Estradiol is known to have a biphasic effect on growth and in high doses initiates the closure of the growth plate (13). It remains to be further investigated whether higher doses of estradiol above +2 SD for girls could further reduce adult height in trans girls as indicated in Boogers et al (25) and in parallel with historical data from treatment trials of tall girls (14, 58, 59).

Overall, we did not see any changes in adult height for the trans boys, in particular no height gain. Almost all reached an adult height within the references for Danish cis girls, which supports the results of the Willemsen et al (26) and Ciancia et al (27) studies. Around half of the trans boys were below −2 SD for Danish cis boys at adult height. No differences in testosterone levels were previously observed between cis boys with short and tall stature (60), which indicates that testosterone levels are not likely to influence adult height. For the small group of trans boys who started HT with a bone age below 14 years, adult height increased but nonsignificantly, by 3.4 cm compared to target height. Thus, bone age at HT start may have an influence on adult height. One study showed a possible augmentation of adult height with oxandrolone treatment when prescribed early in puberty (61), which could potentially be beneficial for those trans boys with a low target height and remaining growth potential.

Our results indicate that trans girls have a greater potential for height reduction when HT is started at a younger bone age. Trans boys may also have a potential for height gain, but both effects need to be investigated further. These changes in adult height could be beneficial for some individuals, as it would improve alignment with their gender identity. We believe that our study can provide knowledge in a field with only a few existing studies. A major limitation of our study is the low number of individuals starting HT early in puberty. Many countries currently introduce age restrictions on HT. However, in the discussion of the long-term consequences of postponing HT until late puberty, when assessing children with long-standing gender dysphoria, our data are of value. Our observational study of a national cohort provides unselected and thus unbiased data on growth and final height. To our knowledge, we are the first to report growth factors during HT for transgender adolescents.

## Conclusion

Our study provides evidence that transgender adolescents starting GnRHa treatment before cessation of puberty or growth reach an adult height within ±2 SD for references for the sex assigned at birth. Effects on adult height in comparison to target height were mostly seen in those trans girls who started HT early. The minor decline in adult height for some trans girls compared to target height, especially after oral estradiol, may be experienced as positive. The differential effect of oral vs transdermal estradiol treatment on IGF-I levels suggests that oral estradiol may be the preferred treatment for tall trans girls.

## Data Availability

The dataset of this study is not publicly available but can be made available from the corresponding author on reasonable request including adherence to legal requirements for confidentiality and data transfer.

## Abbreviations

GnRHa: gonadotropin-releasing hormone agonist
HT: hormone therapy (including both GnRHa and sex steroids)
IGFBP-3: IGF-binding protein-3
PAH: predicted adult height
SDS: SD score
